# Current status on treatment options for feline infectious peritonitis and SARS-CoV-2 positive cats

**DOI:** 10.1080/01652176.2020.1845917

**Published:** 2020-11-13

**Authors:** Aaron M. Izes, Jane Yu, Jacqueline M. Norris, Merran Govendir

**Affiliations:** Sydney School of Veterinary Science, The University of Sydney, Sydney, NSW, Australia

**Keywords:** Cat, feline, feline infectious peritonitis, FIP, feline infectious peritonitis virus, therapeutics, treatment, SARS-CoV-2

## Abstract

Feline infectious peritonitis (FIP) is a viral-induced, immune-mediated disease of cats caused by virulent biotypes of feline coronaviruses (FCoV), known as the feline infectious peritonitis virus (FIPV). Historically, three major pharmacological approaches have been employed to treat FIP: (1) immunomodulators to stimulate the patient’s immune system non-specifically to reduce the clinical effects of the virus through a robust immune response, (2) immunosuppressive agents to dampen clinical signs temporarily, and (3) re-purposed human antiviral drugs, all of which have been unsuccessful to date in providing reliable efficacious treatment options for FIPV. Recently, antiviral studies investigating the broad-spectrum coronavirus protease inhibitor, GC376, and the adenosine nucleoside analogue GS-441524, have resulted in increased survival rates and clinical cure in many patients. However, prescriber access to these antiviral therapies is currently problematic as they have not yet obtained registration for veterinary use. Consequently, FIP remains challenging to treat. The purpose of this review is to provide an update on the current status of therapeutics for FIP. Additionally, due to interest in coronaviruses resulting from the current human pandemic, this review provides information on domesticated cats identified as SARS-CoV-2 positive.

## 1. Introduction

Feline infectious peritonitis (FIP) is a viral-induced, immune-mediated disease with high fatality rates, affecting domesticated cats and some wild felids globally (Addie et al. 2009; Drechsler et al. 2011; Pedersen 2014a). The disease is caused by virulent biotypes of feline coronaviruses (FCoV), known as the feline infectious peritonitis virus (FIPV) (Addie et al. 2009; Drechsler et al. 2011; Pedersen 2014a). FIPV arises through unidentified genetic alterations in FCoV leading to an enhanced capacity to replicate within the monocytes and macrophages (Addie et al. 2009; Pedersen 2009). While FIP can affect cats of any age, its incidence is greatest amongst cats less than three years of age, particularly, those aged between four and sixteen months (Addie et al. 2009; Pedersen 2009). The true prevalence of FIP is not known. While some authors cite that approximately 12%, or one out of nine FCoV-infected cats, will progress to clinical signs of FIP (Addie et al. 2009; Pedersen 2014b), this figure has been extrapolated from veterinary hospital studies, rather than the domesticated feline population at large, and is not congruent with field estimates of the number of cases seen relative to the population. Other studies provide a lower prevalence of FIP. In a case–control study spanning a ten-year period from 1986 to 1995, out of 397,182 accessions presented to North American veterinary medical teaching hospitals, approximately 0.55% of new feline and 0.36% of total feline accessions were cats with FIP (Rohrbach et al. 2001). These FIP patients were significantly more likely to be purebred, young and sexually intact males (Rohrbach et al. 2001; Pesteanu-Somogyi et al. 2006). In a study investigating feline accessions at the North Carolina State University College of Veterinary Medicine over a sixteen-year period from 1986 to 2002, of the 11,535 cats of known breed that were examined, the prevalence of suspected or confirmed FIP in the mixed breed cat population was 0.35% versus 1.3% in the purebred cat population (Pesteanu-Somogyi et al. 2006). For some of these studies, the diagnosis of FIP was made on the basis of clinical examination alone and without confirmatory diagnostic tests (Rohrbach et al. 2001; Pesteanu-Somogyi et al. 2006). In an Australian study, young cats were significantly over-represented amongst FIP cases (Worthing et al. 2012). Domestic crossbred, Persian and Himalayan cats were significantly under-represented compared to the Australian cat population, while several breeds were over-represented, including the British Shorthair, Devon Rex and Abyssinian breeds. A significantly higher proportion of males had FIP (Worthing et al. 2012).

FIP is categorised by its clinical presentation as either a wet (effusive) or dry (non-effusive) form (Addie et al. 2009; Pedersen 2009, 2014a). The wet form is characterised by immune-mediated, fibrinous-granulomatous serositis, often with protein-rich effusions in the thoracic or abdominal cavities (Addie et al. 2009; Pedersen 2009, 2014a). In contrast, the dry form is typified by pyogranulomatous lesions found in multiple body organs and around blood vessels (Addie et al. 2009; Pedersen 2009, 2014a).

FIP-associated mortality is extremely high once clinical signs appear (Pedersen 2014b). As noted by Pedersen (2014b, p.133), ‘*the onset of overt disease is a signal that the cat’s battle with the virus has been lost*.’ Even though some cats can live with FIP for weeks, months or sometimes, years (Pedersen 2014b), survival times generally vary from days to weeks for effusive FIP and weeks to months for non-effusive FIP (Fischer et al. 2011; Tsai et al. 2011; Hugo and Heading 2015). Providing a definitive ante-mortem diagnosis of FIP is challenging, particularly as current diagnostic tests cannot differentiate between FCoV and FIPV (Fischer et al. 2011; Pedersen 2014b). Furthermore, as a confirmed diagnosis relies on positive immunostaining of FCoV antigen by cytology or histopathology, invasive diagnostic tests to collect tissue biopsies might be necessary in sick cats, making confirmation of the diagnosis more challenging at times (Tasker 2018). The reader is directed to recent reviews on more detailed information on the diagnosis of FIP (Drechsler et al. 2011; Pedersen 2014b; Tasker 2018; Kennedy 2020). In reviewing treatment options for FIP, numerous older studies describing potential treatments are mostly based on cases without a confirmed diagnosis of FIP and hampered by the lack of well-controlled clinical trials (Hartmann and Ritz 2008). Likewise, the use of other reported treatment options is currently only supported by *in vitro* studies rather than through *in vivo* clinical studies (Choong et al. 2014; Doki et al. 2016; Hu et al. 2017; Takano et al. 2017). Despite recent antiviral studies with GC376 and GS-441524 showing great promise against FIPV in naturally and experimentally infected cats (Murphy et al. 2018; Pedersen et al. 2018, 2019), these agents have not yet obtained registration for veterinary use (Wogan 2019a, 2019b). Consequently, no effective treatments against FIP are currently legally available to veterinary clinicians (Pedersen 2019a).

## 2. Sars-CoV-2

There is currently great interest in the potential for severe acute respiratory syndrome coronavirus 2 (SARS-CoV-2) to infect animals, including reports of domesticated and zoo-housed wild cats testing positive for this virus (Hosie et al. 2020; United States Department of Agriculture 2020; Wang et al. 2020). The coronaviruses are single strand RNA viruses (Lundstrom 2020) classified within four genera based on genotypic and serological characterisation (*Alphacoronavirus, Betacoronavirus, Gammacoronavirus* and *Deltacoronavirus*; Woo et al. 2012). Whereas FCoV is an *Alphacoronavirus* (Felten and Hartmann 2019), SARS-CoV-2, which causes COVID-19, is a *Betacoronavirus* (Chakraborty and Maity 2020; Lai et al. 2020). Coronaviruses have both a high frequency of recombination and inherently high mutation rates which allow them to adapt rapidly with a heightened potential to infect new hosts (Woo et al. 2006). Viral recombination is mediated in part by the proofreading activity of the nsp14 exoribonuclease, which previously has been shown to hinder the development of nucleoside-based coronavirus treatments (Agostini et al. 2018).

Domesticated cats that have tested positive for SARS-CoV-2 have been associated with SARS-CoV-2 positive owners or suspected SARS-CoV-2 positive owners (Halfmann et al. 2020; Newman 2020). A small number of SARS-CoV-2 positive domesticated cats have demonstrated upper or lower respiratory signs (Sailleau et al. 2020), whereas others have had no overt clinical signs (Newman 2020; Sailleau et al. 2020; Sit 2020; Sit et al. 2020). In limited experimental studies in which large viral inocula are used, it has been reported that cats can transmit SARS-CoV-2 to other cats housed in the same facility via the airborne route (Shi et al. 2020). Another study reported that inoculation and exposure to SARS-CoV-2 leads to nasal shedding in cats and that cats without clinical signs are capable of direct transmission to other cats (Halfmann et al. 2020). Currently, there is no evidence that cats can transmit SARS-CoV-2 to humans (Hosie et al. 2020).

Although there are some antivirals described below that have demonstrated activity against FCoV, it is tempting to extrapolate that they may also reduce shedding of SARS-CoV-2 in rare instances of feline infection. However, to the best of the authors’ knowledge, there are no published *in vivo* studies to verify their SARS-COV-2 antiviral activity in the cat.

## 3. FIP therapeutics

In practice, three major approaches, used either individually or in combination, have been employed to treat FIP (Pedersen 2014b). The first approach seeks to modulate the patient’s immune system non-specifically in order to reduce the clinical effects of the virus through a robust immune response (Pedersen 2014b). The second approach relies on the use of immunosuppressive drugs to dampen the inflammatory response that is central to the pathology in this disease (Hartmann and Ritz 2008; Addie et al. 2009; Pedersen 2014b), whereas the third approach centres on the use of antiviral agents to inhibit viral replication (Murphy et al. 2018; Pedersen et al. 2018, 2019). Although each of these major approaches can be used simultaneously, each will be addressed in turn.

### 3.1. Non-specific immunostimulants

Non-specific immunostimulants have been used as treatments for FIP for many years, often due to unsubstantiated anecdotal reports claiming that such regimens either can improve survival times or serve as an outright cure for FIP (Pedersen 2014b). The over-arching aim of this approach is to encourage the patient to produce an immune response strong enough to reduce the viral load sufficiently to reduce clinical effects of the infection. However, it is somewhat paradoxical that the use of immunostimulants is often paired with immunosuppressive agents, given that some of these drugs may work at cross-purposes to one another (Pedersen 2014b). Examples of non-specific immunostimulants administered as FIP treatments include staphylococcal A protein (Pedersen 2014b), *Propionibacterium acnes* (an immunomodulatory compound derived from gram positive bacteria) (Weiss et al. 1990), lymphocyte T-cell immunomodulators (such as omega [ω] interferon) (Pedersen 2014b) and plant extracts such as polyprenyl immunostimulant (PI) (Legendre and Bartges 2009; Legendre et al. 2017). All of these agents have been unsuccessful or have had limited success as FIP treatments. For example, with respect to the biologic plant extract PI, the use of this agent was originally suggested by Legendre and Bartges (2009), after three cats with the dry form of FIP were reportedly cured after long-term treatment with PI. However, these researches conceded that the treatment had no effect on cats with more severe FIP (Legendre and Bartges 2009). A later field study of PI in 60 cats showed improved survival times with four of these cats surviving over 300 days with an improved quality of life (Legendre et al. 2017). The study also showed that survival times with PI were significantly longer in cats that were not treated with corticosteroids concurrently (Legendre et al. 2017).

### 3.2. Immunosuppressive agents

The use of immunosuppressive agents to dampen the inflammatory response to FIPV stands as something of a therapeutic placeholder. That is, a lack of access to safe and effective alternatives can leave veterinary clinicians with very few options other than to rely on immunosuppressive therapies to control the clinical signs of FIP (Hartmann and Ritz 2008; Addie et al. 2009; Pedersen 2014a). Examples of immunosuppressive agents as FIP treatments include glucocorticoids (e.g. prednisolone, dexamethasone) (Disque et al. 1968; Addie et al. 2009), cytokine inhibitors (e.g. pentoxifylline and propentofylline) (Fischer et al. 2011) and alkylating agents (e.g. cyclophosphamide and chlorambucil) (Bilkei 1988; Addie et al. 2009). Although glucocorticoid administration reduces clinical signs in cats with FIP, there is no evidence that they are curative for infected cats (Addie et al. 2009). Claims that agents such as glucocorticoids are effective against FIP have been disproved by placebo-controlled, double blinded studies by Ritz et al. (2007) and Fischer et al. (2011). In these studies, investigating the effects of feline interferon ω (Ritz et al. 2007) and propentofylline (Fischer et al. 2011) on the survival time and quality of life of FIP-affected cats, all cats (including those in a placebo group) received dexamethasone and/or prednisolone. The respective authors reported that there was no significant difference in survival times between those FIP-affected cats that received either feline interferon ω (Ritz et al. 2007) or propentofylline (Fischer et al. 2011) (median survival time of nine days for feline interferon ω and eight days for propentofylline) and those in the control group administered glucocorticoids only (median survival time of eight days in both studies). Given the co-administration of dexamethasone and/or prednisolone in both of these studies, it is difficult to determine the effects of feline interferon ω or propentofylline as a single therapeutic agent. Feline interferon ω has been shown to inhibit FCoV replication *in vitro* (Mochizuki et al. 1994). An uncontrolled trial with feline interferon ω and glucocorticoids yielded a promising result with 67% of cats achieving complete or partial remission, but FIP was not confirmed in these cases (Ishida et al. 2004). When recombinant human leukocyte alpha interferon or feline fibroblastic beta interferon were used alone, neither reduced the mortality in treated cats compared to controls (Weiss and Toivio-Kinnucan 1988; Bolcskei and Bilkei 1995; Ritz et al. 2007). However, when a high dose of alpha interferon was used in combination with *Propionibacterium acnes,* the mean survival time was prolonged, but only by three weeks (Weiss et al. 1990). Overall, although interferons are frequently used in cats with FIP, their efficacy is questionable. Likewise, the use of cyclophosphamide also has been investigated in cats with FIP in combination with prednisolone and ampicillin (Bilkei 1988). Seventy-six of 151 cats were regarded as ‘healthy’ after therapy. However, cats included in this study had no confirmed diagnosis of FIP. Another study investigating the use of cyclophosphamide in addition to prednisolone and ampicillin in suspected FIP cases reported that 29–80% of cats died in three years (Bolcskei and Bilkei 1995). Again, FIP was not confirmed in these cats. Ozagrel hydrochloride (a thromboxane synthesis inhibitor) has also been used as an immunosuppressive agent against FIP (Watari et al. 1998). Although it was shown to have a beneficial effect in two cats, FIP was not confirmed in either of these patients (Watari et al. 1998).

### 3.3. Specific FIP therapeutics

The third approach to the treatment of FIP involves the administration of antiviral agents to target either the cellular mechanisms that viruses co-opt for replication, or alternatively, a specific aspect of virus activity related to infection and/or replication (Hartmann and Ritz 2008; Addie et al. 2009; Pedersen 2014a). Antiviral drugs that inhibit FCoV have been identified but many have not been successfully trialled in infected patients (Weiss et al. 1993; Hartmann and Ritz 2008; Addie et al. 2009; McDonagh et al. 2014; Pedersen 2014b). However, a new therapeutic breakthrough using the nucleoside analog GS-441524 as a direct acting antiviral drug for FIP has been reported (Murphy et al. 2018; Pedersen 2019a, 2019b; Dickinson et al. 2020). GS-441524, the active metabolite of remdesivir, is an RNA-chain terminator of viral RNA dependent RNA polymerase (Murphy et al. 2018; Pedersen et al. 2019) and has been found to strongly inhibit FIPV both in tissue culture and experimental cat infection studies as well as in cases of naturally occurring FIP (Murphy et al. 2018; Pedersen et al. 2019). Utilising an *in vitro* approach, Murphy et al. (2018) determined that GS-441524 was non-toxic in Crandell Rees feline kidney (CRFK) cells at 100 µM concentrations whilst still being able to inhibit FIPV replication in both cultured CRFK cells and naturally infected feline peritoneal macrophages at 1.0 µM concentrations. In a companion *in vivo* study in cats experimentally infected with FIPV (serotype I FIPV m3c-2 strain), GS-441524 was administered once a day (5.0 mg/kg BW or 2.0 mg/kg BW as a subcutaneous injection [SC]) for two weeks once the disease course became established (Murphy et al. 2018). Requiring at least two weeks of treatment, this regimen led to a rap id reversal of clinical signs and a return to normality in all subjects. No toxicity was noted. Building on this work, Pedersen et al. (2019) investigated the *in vivo* therapeutic effects of GS-441524 on naturally occurring FIP, including both the wet and dry forms of the disease. In this study, 31 cats (26 with effusive FIP and five with non-effusive FIP) were recruited and administered a dosage of 2.0 mg/kg BW SC once daily for at least 12 weeks. The dosage was increased to 4.0 mg/kg BW SC once daily when indicated by deteriorating clinical signs. Four cats were euthanised or died within the first five days of the experiment as a result of the severity of their infection. A fifth cat was euthanised after 26 days due to a lack of treatment response. The remaining 26 cats successfully completed the twelve-week (or longer) trial. Eighteen of the cats remained healthy after one course of treatment whereas the remaining eight suffered relapses within 3 to 84 days. Three of the eight relapsing cats were treated again at the same dosage whilst five cats had the dosage increased from 2.0 to 4.0 mg/kg BW. The five cats treated with the higher dose remained healthy. Of the remaining three cats treated at the original lower dose, two of them relapsed a second time and required a third treatment with the higher dose. These two cats remained healthy after the second dose. The third cat relapsed after a second round of the lower dose and was euthanised due to the severity of its neurological disease. Ultimately, the study produced 25 long time survivors. Injection site reactions were the most common side effect (Pedersen et al. 2019). Based on these findings, the authors concluded that GS-441524 is a safe and effective treatment for FIP when administered at a dosage of 4.0 mg/kg BW SC once a day for at least 12 weeks (Pedersen et al. 2019). A higher dosage of 5.0 to 10.0 mg/kg BW SC once a day for 12 weeks has been recommended for neurological FIP cases as determined by the treatment of four clinical cases (Dickinson et al. 2020). Given the consistency of its reported efficacy, a burgeoning global black-market for GS-441524 has arisen since it has not been formally approved for commercial use anywhere (Pedersen 2019b). Interestingly, GS-441524 has also displayed *in vitro* antiviral activity against SARS-CoV (Cho et al. 2012).

Likewise, remdesivir (GS-5734), a prodrug of the parent adenosine nucleoside analog, GS-441524 (Amirian and Levy 2020), has been granted emergency use authorisation by the U.S. Food and Drug Administration (F.D.A.) to treat suspected or laboratory-confirmed COVID-19 in adults and children hospitalised with a severe infection. This emergency authorisation was based on a randomised, double-blinded, placebo-controlled trial conducted by the National Institute of Allergy and Infectious Diseases (NIAID) (NCT04280705) and a sponsored open-labelled trial that evaluated different durations of remdesivir (NCT04292899). Another randomised, double-blinded, placebo-controlled, multicentre trial conducted in China also showed that the use of remdesivir in adult patients with COVID-19 was associated with a reduction in time to clinical improvement when treated early, although statistically significant clinical benefits were not observed (Wang et al. 2020). Clinical trials of remdesivir and many other antivirals are currently underway in many countries for the treatment of COVID-19 (Amirian and Levy 2020).

Whilst not related to the direct treatment of FIP, a medication proposed for reducing FCoV may be a likely precursor to FIP prevention. An antiviral product called Mutian® X, a synthetic adenosine analogue, whose exact nature is a ‘commercial secret’, has been shown to stop faecal feline coronavirus shedding in chronically infected cats when administered orally at 4.0 mg/kg BW, once daily for four days (Addie et al. 2020). Addie et al. (2020) suggested that a combination of probiotics and interferon may have reduced feline coronavirus shedding in two cats in the same study. Feline interferon ω has been shown to reduce viral excretion of feline coronavirus in retrovirus infected cats (Gil et al. 2013).

### 3.4. Other antiviral compounds

Other candidate compounds have also been trialled for their antiviral activity against FIPV. For example, the antiviral ribavirin was tested *in vivo* in cats experimentally infected with FIPV and found to possess marginal antiviral activity against FIPV and toxicity to cats (Weiss et al. 1993]. Cyclosporin A, a cyclophilin inhibitor, has been shown to inhibit replication of feline coronavirus *in vitro* although the mechanism of its inhibitory effects is unknown (Pfefferle et al. 2011; Tanaka et al. 2012, 2013). Cyclosporin was administered to one cat with effusive FIP and a reduction in pleural fluid and viral load was observed after treatment. The cat died of respiratory failure on day 264 but the cause of death was not determined (Tanaka et al. 2015). Likewise, two compounds, galanthus nivalis agglutinin (GNA) and nelfinavir (a protease inhibitor) when used in combination were able to inhibit FCoV replication *in vitro* (Hsieh et al. 2010). Yet, ultimately, neither of these compounds was found effective when tested under conditions simulating FIPV-infection. Similarly, based on promising findings concerning 3 C-like protease inhibitors efficacy against FCoV (Kim et al. 2013, 2015, 2016), a field trial of GC376 was undertaken with 20 cats with various forms of FIP, excluding those with neurological signs (Pedersen et al. 2018). Cats were administered a dosage of 15.0 mg/kg BW, SC, twice a day for a minimum of 12 weeks. Whilst results were encouraging (with seven cats achieving a mean disease remission of 11.2 months), side effects developed, including transient pain upon injection, subcutaneous fibrosis, alopecia and abnormal development of permanent teeth in cats treated before 16–18 weeks of age (Pedersen et al. 2018). Other protease inhibitors have been shown to target the 3 C-like protein of coronaviruses (Rathnayake et al. 2020; Theerawatanasirikul et al. 2020).

The antifungal itraconazole has demonstrated *in vitro* anti-FIPV activity at low drug concentrations (2.5 µM) (Takano et al. 2019). A recent *in vivo* study investigated the effects of a combination of itraconazole (50 mg/animal per os, once a day) and an anti-human TNF-alpha monoclonal antibody (10 mg/animal) for the treatment of three cats experimentally infected with FIPV (Doki et al. 2020). Although two of the three cats showed improvement from FIPV-related clinical signs, an increase in the peripheral blood lymphocyte count and a decrease in alpha-1-acid glycoprotein were identified after treatment began. The third cat was euthanised due to its failure to respond to treatment. The authors concluded that this combination of drugs may be useful until more effective anti-FIPV agents become available. Itraconazole, in combination with prednisolone, have been used to treat effusive FIP in a three-month-old male Scottish Fold kitten, leading to a reduction in pleural effusion (Kameshima et al. 2020). However, this cat showed neurological manifestations and was euthanised due to status epilepticus after 38 days of treatment (Kameshima et al. 2020). Moreover, other agents have also been shown to have *in vitro* antiviral activity against FCoV, including an anti-feline TNF-alpha monoclonal antibody (Doki et al. 2016); U18666A (a cholesterol transport inhibitor) (Takano et al. 2017; Doki et al. 2020); diphyllin (a vacuolar ATPase blocker) and its nanoformulation (Hu et al. 2017) and a circular triple helix forming oligonucleotide RNA (Choong et al. 2014). However, no *in vivo* studies have been reported using these compounds.

The human antimalarial drug chloroquine also inhibits FIPV replication *in vitro* (Takano et al. 2013; McDonagh et al. 2014). Chloroquine has long been known to possess both anti-inflammatory and *in vitro* antiviral properties against a diverse range of viruses (Takano et al. 2013). Yet, despite its good *in vitro* efficacy, chloroquine displayed poor antiviral efficacy against *in vivo* experimentally induced FIP infection (Takano et al. 2013). In their study, Takano et al. (2013) found that chloroquine treatment was associated with an improvement in clinical scores and a slightly increased, but not statistically significant, survival time of cats infected with the highly virulent FCoV FIPV1146. Additionally, increased activity levels of alanine aminotransferase in the chloroquine-treated groups indicated a potential problem with hepatic damage. The dosage of 10 mg/kg BW twice weekly SC was extrapolated from human dosing protocols and not based on any known pharmacokinetic studies in cats. Recently, hydroxychloroquine’s antiviral activity against FIPV has also been studied *in vitro*. When used with recombinant feline IFN-ω, hydroxychloroquine showed increased antiviral activity against FIPV infection (Takano et al. 2020). Hydroxychloroquine has also been investigated in clinical trials for COVID-19 treatment in people (Lundstrom 2020). However, this drug has resulted in controversial results, leading to the current conclusion that its clinical efficacy in patients with COVID-19 has not been verified to date (Lundstrom 2020).

McDonagh et al. (2014) screened 19 candidate compounds used in the treatment of other coronavirus infections for their cell toxicity and effectiveness at inhibiting FIPV replication in infected CRFK cells. In this *in vitro* study, a resazurin-based cytopathic effect (CPE) inhibition screening test was developed to investigate antiviral efficacy against two strains of FCoV: FECV 1683 and FIPV 1146. Other assays, including plaque reduction, virus yield reduction and viricidal suspension were used to evaluate the antiviral effects of the candidate compounds. Ultimately, the study identified three compounds (chloroquine, mefloquine and hexamethylene amiloride) that substantially reduced the viral load of FIPV in infected CRFK cells without cytotoxic effects at 10 µM concentrations. Moreover, preliminary experiments suggested that the antiviral mechanisms of all three compounds acted at an early stage of viral replication.

Given the potential impact of these results for the treatment of FIP, further investigation of these compounds is warranted (McDonagh et al. 2014). Both chloroquine and mefloquine are commercially available pharmaceutical agents registered for use in people. Each has an extensive body of supporting literature regarding their pharmacokinetics and safety in non-feline species and some information is now available on mefloquine’s pharmacokinetic profile in cats (Izes 2019; Izes et al. 2020a, 2019, 2020b; Yu et al. 2020). Considerably less is known about hexamethylene amiloride, particularly with respect to its safety (McDonagh et al. 2014). As Takano et al. (2013) previously discounted the *in vivo* efficacy of chloroquine as an antiviral agent for FIP, clinical trials of mefloquine to treat infected cats could be considered. Recent *in vitro* pharmacokinetic studies have indicated that mefloquine undergoes phase I hepatic metabolism, but not phase II glucuronidative metabolism, when catalysed by feline hepatic microsomes and therefore is not likely to have delayed elimination in cats (Izes et al. 2020a). Mefloquine’s pharmacokinetic profile in cats has been investigated in anticipation of undertaking clinical trials to inhibit feline coronavirus and feline calicivirus in those cats clinically affected by these viral infections (Yu et al. 2020). Further research on the clinical efficacy of mefloquine in FIP-confirmed cats is underway.

With respect to all these antiviral agents, it is important to note that a combination of therapeutics may be required to be administered to patients to inhibit monotherapy selection of viral resistance.

## 4. Conclusion

Despite the absence of their commercial availability, antivirals such as GS-441524 and GC376, have been shown to be efficacious treatment options against FIP. Whilst pending the regulatory approval of these agents, other commercially available agents, such as mefloquine and itraconazole, need to be investigated more thoroughly through *in vivo* studies to ascertain their true potential as FIPV antivirals. Moreover, whilst to date, the current SARS-CoV-2 pandemic has had little reported impact on veterinary medicine, a global One Health approach dictates that a vigilant watch is kept on therapeutic advances in the fight against coronaviruses as the information may ultimately be useful across species.
